# Effectiveness of Early Physiotherapy Rehabilitation Approach for Split Skin Grafting Post-burn in a Pediatric Patient

**DOI:** 10.7759/cureus.44083

**Published:** 2023-08-25

**Authors:** Anushka Raipure, Shubhangi Patil, Heena Pathan

**Affiliations:** 1 Community Health Physiotherapy, Ravi Nair Physiotherapy College, Datta Meghe Institute of Higher Education & Research, Wardha, IND

**Keywords:** pediatric burn pain management, split-thickness skin graft, physical rehabilitation advance research interest, pediatrics rehabilitation, post operative rehab and soft tissues injuries

## Abstract

Childhood is critical for developing social, physical, and cognitive functioning. Burns in children are very different from burns in adults because it is difficult for them to save themselves. Their skin is more sensitive and less heat-resistant; their exposure may last longer, worsening the burn. When neglected, severe disabling and deforming contractures caused by burns in children can result in substantial impairment. Therefore, burn rehabilitation should not be carried out by one person but rather by a multidisciplinary team to ensure that all of the patients' physical, psychological, and social requirements are met while the child is in the hospital and after discharge. The treatment of young burn victims requires a multidisciplinary approach that begins the day of the accident and lasts for several years. To reduce the consequences of the patient's post-traumatic stress and increase functional independence, a thorough rehabilitation programme is needed. The burn team members' dedication, commitment to the patient's care, and encouragement of patient participation and full engagement in rehabilitation can make a difference to juvenile burn patients' long-term quality of life (QOL). We present a seven-year-old female who suffered from a superficial burn over the lateral aspect of her right thigh. Exercise prescriptions should be thoroughly customized to provide the best possible success in rehabilitation, considering the degree of burns and physical limitations. This case report is intended to serve as a practical manual for the necessary clinical knowledge and therapy intervention approaches for managing burn patients successfully.

## Introduction

Childhood plays a crucial role in developing social, physical, and cognitive abilities. Unfortunately, burn injuries are relatively common, especially among children worldwide. These burns can have a lasting impact, often starting during childhood and continuing into adulthood [1]. Newly available data reveals that burn injuries lead to the hospitalization of more than 500,000 children annually, which is particularly prevalent in economically disadvantaged nations. These incidents give rise to significant consequences, including substantial challenges in terms of function, social integration, and psychological well-being. It is crucial to emphasize that burn injuries present differently in children than adults due to inherent physiological and behavioral differences. Their limited mobility and cognitive development hinder children's ability to remove themselves from the source of the burn. Additionally, their skin is more sensitive and less heat-resistant, potentially extending the exposure duration and worsening the burn injury's severity [2].

Moreover, children are particularly vulnerable due to their smaller body size and higher surface area-to-weight ratio. Neglected burn injuries in children can result in significant impairments, including severe and deforming contractures. Consequently, effective burn rehabilitation requires a multidisciplinary team approach, addressing the child's physical, psychological, and social needs during hospitalization and post-discharge [2]. Burn injuries contribute significantly to avoidable illness and mortality rates. Burn injuries, mainly when occurring in childhood, can give rise to persistent disabilities, encompassing diminished mobility, structural deformities, and sensory impairments. These disabilities influence routine activities, contribute to prolonged discomfort, and precipitate psychological distress, including compromised self-esteem and social isolation. The imperative for effective rehabilitation becomes evident in light of these ramifications, as it facilitates these outcomes, fosters functional self-sufficiency, tackles psychological complexities, and elevates overall life quality [3].

Burn injuries have local and systemic effects, characterized by a complex pathophysiologic process. Patients with burn injuries covering at least 30% of their total body surface area (TBSA) often experience systemic symptoms due to releasing cytokines and catecholamines. The initial stage, previously referred to as "burn shock," results from fluid third-spacing, leading to hypovolemia [4]. Hyperdynamic circulation, high oxygen consumption, and a hypermetabolic phase follow, beginning 24 hours after the injury and regulated by cortisol, catecholamines, and cytokines [5]. Given the considerable morbidity and mortality associated with both short- and long-term effects of burn injuries, comprehensive care at all stages of a child's treatment is crucial. The subsequent sections delve into the impact of these physiological changes on the perioperative management of these patients [6].

Effective pain management for pediatric burn patients is an evolving and promising field, with non-pharmacological approaches gaining traction. Open communication, involvement, and medical play are employed in child life therapy to reduce pain and anxiety scores significantly [7]. Distraction techniques, including tablet-based interventions, have successfully lowered anxiety levels before, during, and after hydrotherapy sessions [8]. Virtual reality (VR) has emerged as a practical method to reduce anxiety and pain. A recent meta-analysis of six randomized trials on VR in burn care found that four demonstrated a statistically significant reduction in pain during burn procedures [9]. Given the advancements in burn care and survival rates, managing pain and anxiety in these vulnerable pediatric patients is paramount, as it profoundly affects their short- and long-term outcomes [10]. Up to 38% of hospitalized pediatric burn victims develop anxiety disorders, with pain being a recognized contributor to post-traumatic stress disorder (PTSD) and subsequent acute stress symptoms [11]. Exploring alternative modalities and relatively underutilized pharmacological agents shows promise for pain management. It is imperative to aggressively, creatively, and collaboratively pursue pain management research and protocols within the pediatric burn population to enhance inpatient recovery and mitigate long-term psychological effects [12].

Caring for young individuals who have suffered burn injuries requires a thorough and collaborative strategy that commences shortly after the incident and endures over an extended period. Implementing a comprehensive rehabilitation regimen is vital to mitigating the repercussions of post-traumatic stress and promoting increased functional self-sufficiency. The unwavering devotion and determination exhibited by the burn-care team, coupled with its active facilitation of patient involvement and commitment to the rehabilitation process, wield a substantial impact on the enduring quality of life (QOL) experienced by young burn survivors [13].

We present the case of a seven-year-old female who sustained a superficial burn on the lateral aspect of her right thigh.

## Case presentation

**Figure 1:**
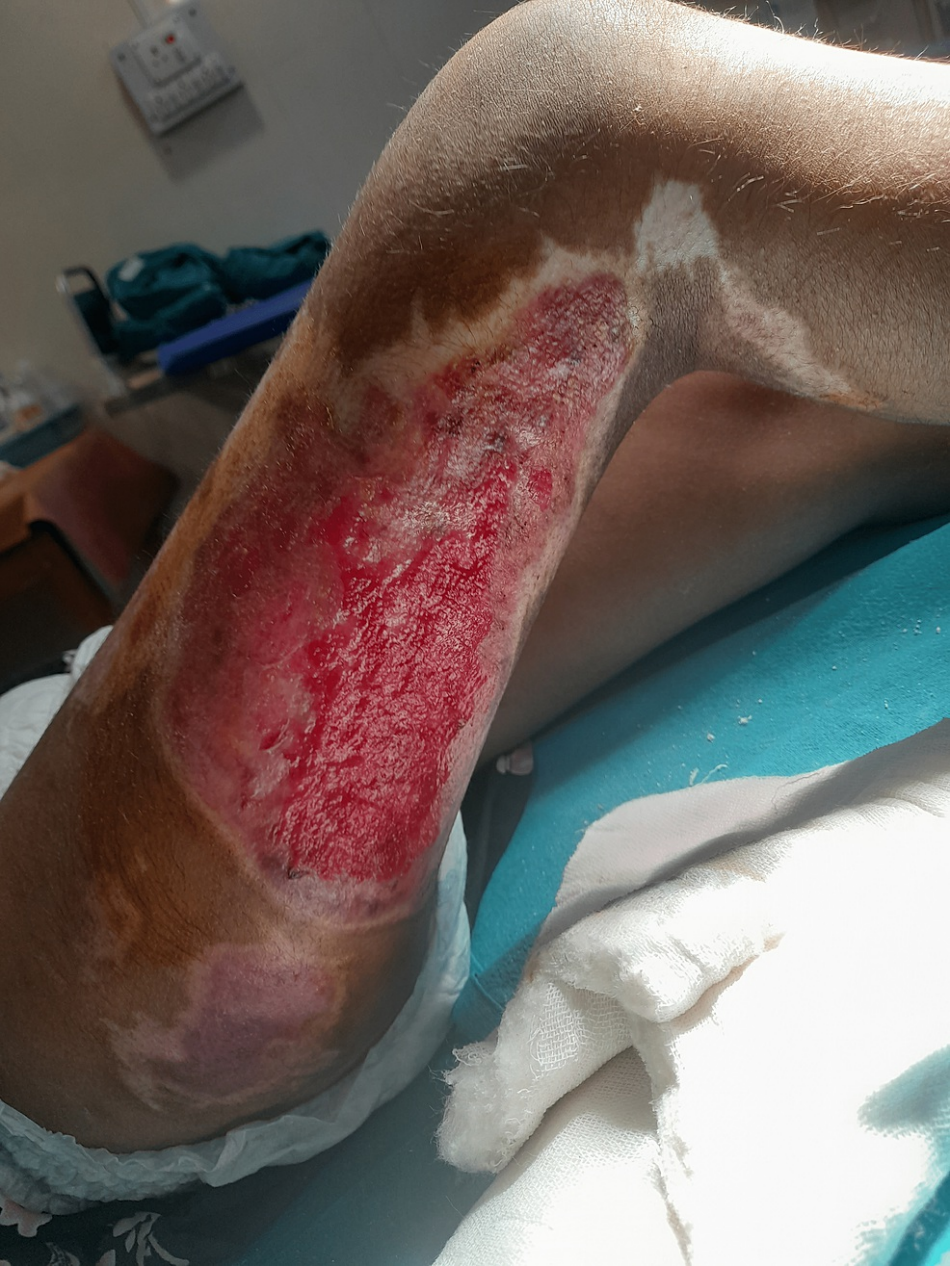
Burn wound before surgery

**Figure 2:**
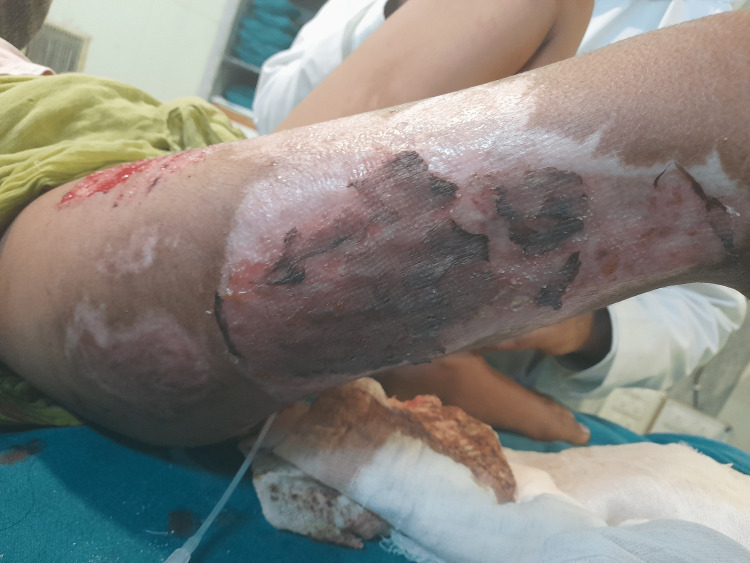
Post-operative split skin grafting

A seven-year-old girl was brought by her mother to the burns unit of the tertiary care Acharya Vinoba Bhave Hospital, Sawangi, Maharashtra, India. She suffered a superficial burn injury over right thigh and partial thickness burn over distal part of thigh caused by her clothes catching on fire. The burns covered 4% of the body surface area. The patient had undergone split skin grafting of right thigh. She was then referred to physiotherapy for further management. Pre-physiotherapy assessments are shown in the table below. Figures 1 and figure 2 show pre- and post-operative pictures of burn wound of the patient.

Burn injury physical and occupational rehabilitation programmes often concentrate on regaining and preserving the range of motion, minimizing scarring, and avoiding contractures [14]. It's important to carefully assess several post-burn physical and functional limitations that can prevent burn victims from achieving independence. Home management, work responsibilities, and leisure activities are all restricted because of the constraints on the range of motion, mobility, intolerance for standing or walking, discomfort, and diminished strength and endurance [15]. The legal guardian's informed consent was obtained on the first day of the acute admission. During the initial phases of the treatment, the grafting locations severely restricted the use of traditional physiotherapy methods. Table 1 shows interventions administered.

**Table 1:** Intervention protocol designed for the patient

Exercise	Dosage
Patient and caregiver education	The therapist maintains a consistent effort to offer ongoing education and support to both patients and care providers throughout every phase of recovery, starting from admission and extending to scar management. The therapist tailors the education provided to align with the patient's age, cognitive development, psychological state, as well as the circumstances of their family and caregivers. This proactive approach guarantees a comprehensive and personalized level of support during the entire recovery process.
Pain management	Distraction using mobile phone virtual reality (VR) game [16], relaxation techniques
Edema Management	Implementing passive, active-assisted, or active range-of-motion exercise programs suited to the level of arousal or pain throughout all phases of healing and rehabilitation
Contracture Management	Initially, the patient was made to perform static quadriceps and hamstrings with a five-second hold and then was given active assisted range-of-motion movements to the hip and the knee.
Respiratory Management	We reduced bed rest by limiting immobility. Deep breathing exercises. Since the patient complained of cough initially, postural drainage techniques were employed, i.e., percussion and vibrations. Scapulothoracic and upper limb mobility exercises.
Gait Training	We facilitated sit-to-stand maneuvers by using a bolster, combining them with single-leg sit-to-stand exercises and multidirectional reach-outs while the child was seated. During standing training sessions, the child actively participated in tasks, receiving substantial support at the pelvis and knees. After an additional two-week period, we gradually assisted her in taking a few steps, focusing on minimizing pelvic support. We introduced task-oriented strategies to promote effective weight shifting. Gait training followed, with a gradual reduction in pelvic support assistance. The activities included stepping forward, accompanied by reach-outs. We further progressed to step-up and step-down exercises, incorporating stepper, bolster, and half-kneeling techniques. Critical components of balance training include walking training on a stepper and walking between parallel bars in all directions while facing a large mirror. During gait training, rolls and wedges are openly positioned obstacles across the walking course. Additionally, walking exercises are performed on various surfaces, including a soft mat, sponge, carpet, or hard surface.
Aerobic Exercise	Engage in exercise sessions with a frequency of three to five days per week. Adjust the intensity to encompass 70%–85% of your peak aerobic capacity (VO_2_ peak) or surpass 80% of your maximum heart rate (HR peak). Achieve this by partaking in either treadmill routines utilizing one- or two-minute intervals or cycle ergometer activities employing two-minute intervals. Allocate 20–40 minutes for the main workout, excluding warm-up and cool-down periods. This duration can be utilized for continuous exercise or work intervals followed by rest or moderate activity periods. Diversify your exercise modes by selecting from various options, including treadmills, cycle ergometers, elliptical machines, arm ergometers, and rowing machines, and also incorporating sports such as soccer, basketball, and kickball. As you progress, gradually increase your daily physical activity to reach a target of 5,000–10,000 steps over 12 weeks. Strive to accumulate more than 150 minutes of aerobic exercise per week. Initiate sessions at a duration of 10 minutes per day, and over the 12 weeks, progressively extend this to a 60-minute daily session, encompassing warm-up and cool-down phases [14].
Resistance Exercise	To optimize effectiveness, individuals should engage in exercise sessions two–three days per week; however, individuals can explore the option of daily workouts by alternating between upper and lower body routines. Regarding intensity, repetitions, and sets, it is advisable to follow a progressive approach. During the initial one–two weeks, aim for an intensity level equivalent to 50%–60% of the three repetition maximum (3RM), targeting a repetition range of 12–15. As the routine progresses into weeks three–six, elevate the intensity to 70%–75% of the 3RM, focusing on a repetition range of 8–10. For the subsequent weeks, specifically weeks 7–12, maintain a consistent intensity of 75–85% of the 3RM and a repetition range of 8–12. Complete two–three sets for each exercise. Give careful consideration to the sequence of exercises as well. Start the routine with exercises targeting larger muscle groups before transitioning to those engaging smaller muscle groups [14].

**Figure 3:**
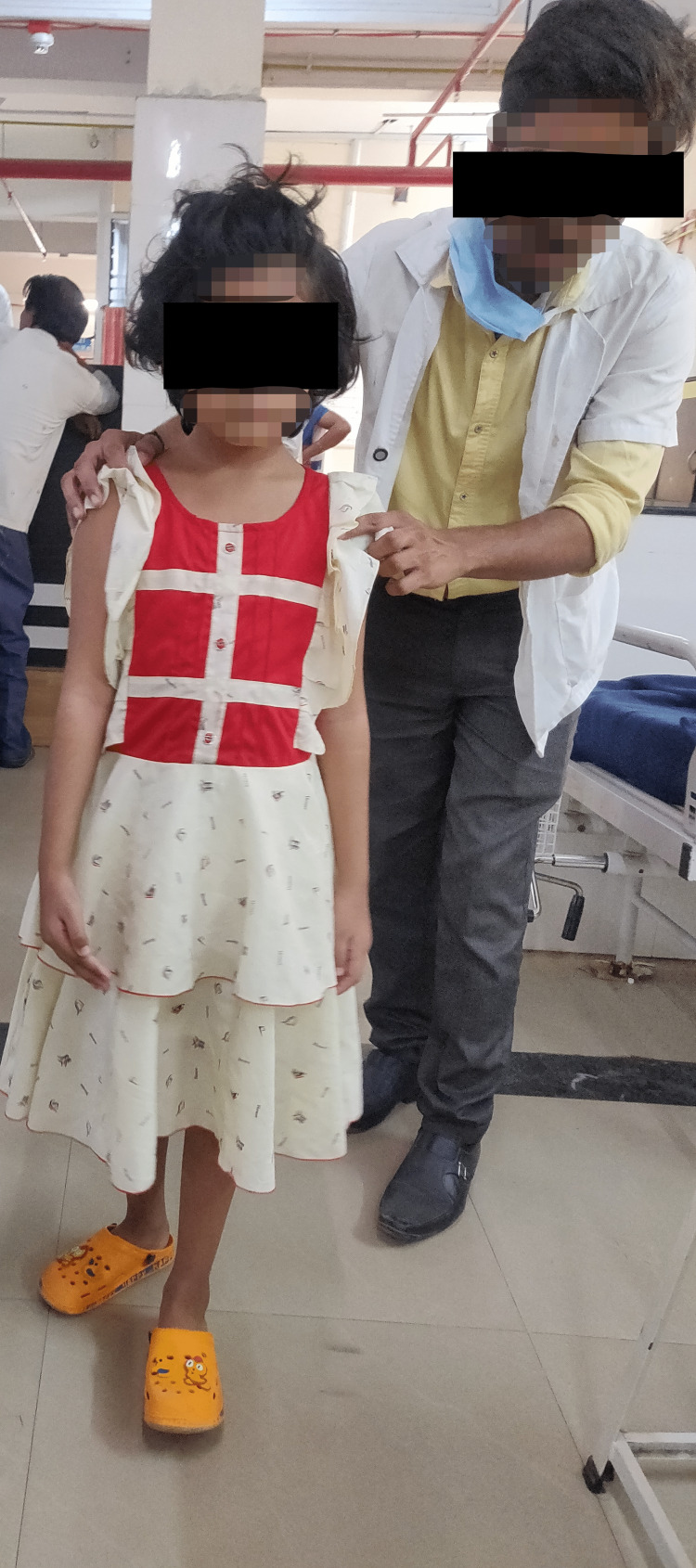
Gait training sessions of the patient

**Figure 4:**
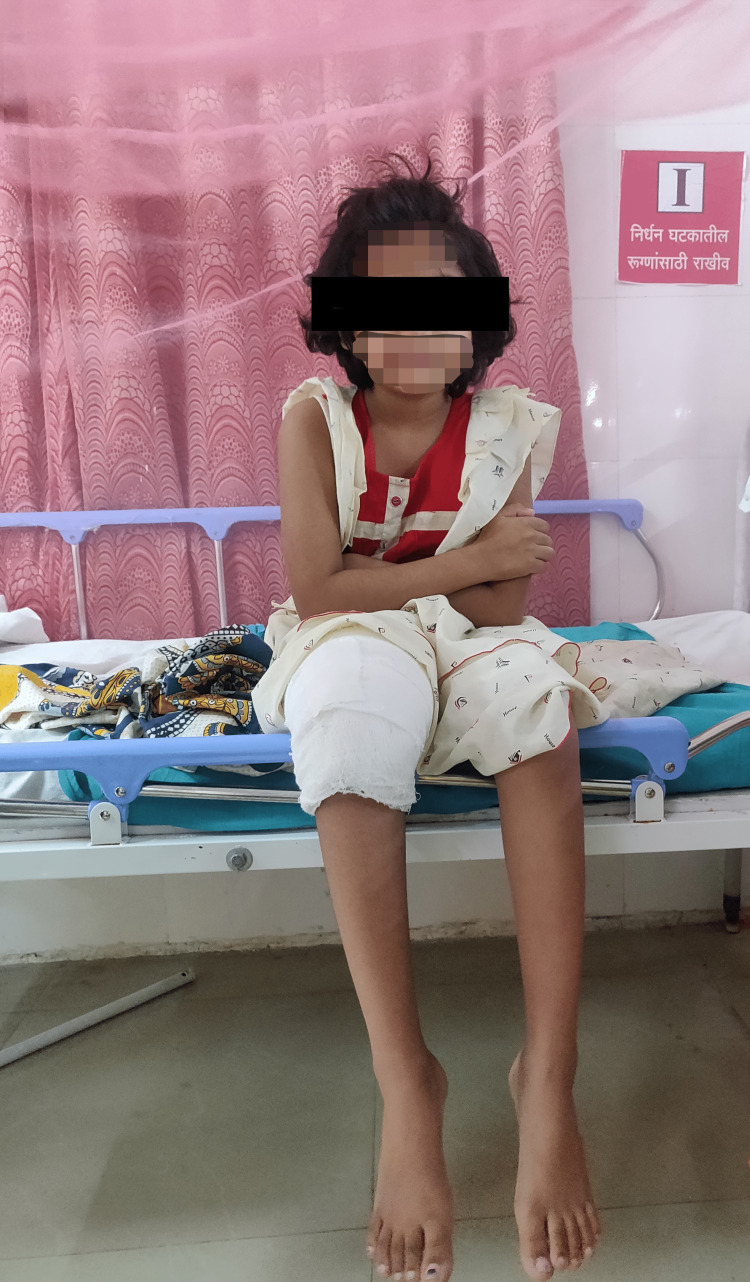
Patient sitting without support

Figures 3 and 4 show training sessions of the patient.

Ongoing rehabilitation is necessary to guarantee that patients and their families have functional recovery and social reintegration after leaving a hospital setting. A more homelike and pleasant environment outside of the hospital can help patients continue improving with the help of effective discharge planning. Physical difficulties after treatment and rehabilitation in the hospital are particularly difficult for children. It is widely acknowledged that exercise therapy-based programmes can significantly affect children's physical and emotional well-being [16]. Table 2 shows pre- and post-outcome measures.

**Table 2:** Pre- and post-outcome measures

Outcome Measures	Pre-intervention	Post-intervention
Faces pain rating scale	10 (hurts worst)	0 (No Hurt)
Range of motion	Right	Right
Hip flexion	N/A	0-135
Hip extension	N/A	0-20
Hip abduction	N/A	0-40
Hip addcution	N/A	40-0
Hip external rotation	N/A	0-40
Hip internal rotation	N/A	0-40
Knee flexion	N/A	0-140
Knee extension	N/A	140-0
Manual muscle testing	Right	Right
Hip flexors	N/A	4/5
Hip extensors	N/A	4/5
Hip abductors	N/A	4/5
Hip addcutors	N/A	4/5
Hip external rotators	N/A	4/5
Hip internal rotators	N/A	4/5
Knee flexors	N/A	4/5
Knee extensors	N/A	4/5
Pediatric balance scale	20/56	52/56
WeeFIM	Level 2 maximal assistance	Level 7 complete independence
Strengths and difficulties questionnaire		
Total difficulty score	Boderline	Normal
Emotional symptoms score	Borderline	Normal
Conduct problem score	Normal	Normal
Hyperactivity score	Normal	Normal
The Zarit Caregiver Burden Scale	Moderate burden	Minimal burden

## Discussion

In low-income nations, burn injuries are a common reason for hospital admission, and they frequently result in secondary problems like scar tissue formation, deformity, and contractures [17]. The acute, chronic, and rehabilitative phases can all benefit from improved functioning and participation, which can be achieved through physiotherapy. It is crucial to determine which rehabilitation and physiotherapy approach successfully restore function in contexts with limited resources and access to adequate therapy [18].

Burns are a significant source of lost disability-adjusted life years (DALYs) in low- and middle-income countries and are a global public health issue. Burn injuries include a wide range of morbidities, and they frequently leave both physical and psychological scars. In the burn unit, survivors of high-voltage electrical injuries are responsible for many amputations and lengthier periods of stay. Damages that result in post-burn issues are incredibly stressful, are linked to functional impairments, and can make it difficult to return to work and do other daily tasks [19]. Adopting an aggressive strategy for handling such patients with rehabilitation starting from day one could significantly improve the development of post-burn problems, frustration, and stress of a juvenile burn child and parent(s). Even though it is a widely acknowledged ideal in burn care, patient-centred care remains one of the biggest obstacles [20].

In a burn patient, mobilization and exercise should start as soon as possible. Only passive interventions, such as splinting and positioning, should be started in the acute stages to reduce the likelihood of burn scar contracture. Active exercise should begin as soon as the patient is stable to support function maintenance and address the hypermetabolic state.

We followed a similar protocol along with the stump care regimen for the residual limb. Additionally, care was taken to avoid the occurrence of hypertrophic scarring, which is the most typical consequence following burn injury (67% prevalence). We increased the patient's neck and shoulder range of motion and stopped the emergence of new issues by adhering to this rigorous rehabilitation programme [21]. Early physical therapy reduces functionally debilitating scar contractures while maintaining joint mobility, promoting oedema resolution, preventing muscular atrophy, disusing osteoporosis, and respiratory and cardiac problems [22].

Palacick et al. conducted a cross-over study aimed at comparing the efficacy of the six-minute walk test and the modified Bruce treadmill test in assessing cardiovascular functional capacity in pediatric patients with severe burns [14]. The study's findings revealed that the modified Bruce treadmill test induces a significantly greater challenge to the cardiorespiratory system compared to the six-minute walk test. This distinction was evident through measurements of maximum heart rate (HR) as well as the patient's perceived exertion, as quantified by the rate of perceived exertion. The study suggests that, when feasible, the utilization of the modified Bruce treadmill test is recommended for a more comprehensive evaluation of cardiovascular functional capacity. However, it is worth noting that the six-minute walk test holds greater clinical feasibility when assessing pediatric patients with burns. Notably, this alternative test offers valuable insights into submaximal functional exercise capacity. In conclusion, the study underscores the advantages of the modified Bruce treadmill test for robust cardiovascular assessment, while acknowledging the pragmatic benefits of the six-minute walk test, particularly in the context of pediatric patients with burns [14].

After experiencing a severe burn injury, the body initiates a systemic stress response that triggers metabolic and inflammatory disturbances that leads to the loss of muscle mass, commonly called muscle wasting. This phenomenon persists for months or years, and is exacerbated by prolonged disuse periods. The equilibrium between muscle protein synthesis and breakdown, essential for maintaining muscle mass, is intricately governed by complex signaling pathways. However, these intricate pathways following burn injuries culminate in an overall negative protein balance within the skeletal muscles. The current understanding of these molecular mechanisms needs to be more comprehensive and conclusive, impeding the development of effective therapeutic interventions. A comprehensive review by Dombrecht et al. consolidates our understanding of the molecular underpinnings driving muscle protein balance [23]. It evaluates the potential of exercise as a promising strategy to counteract the detrimental muscle effects post-burn. To establish practical therapeutic approaches, it is imperative to ascertain which specific molecular mechanisms contribute to muscle wasting. Furthermore, this review underscores the need for dedicated research to investigate the distinct impacts of exercise on these intricate pathways. This pursuit holds the key to confirming the viability of exercise-based interventions and setting the groundwork for targeted therapeutic modalities for individuals recovering from burn injuries [23].

Ali et al. conducted a study to evaluate the immediate impact of integrating VR into conventional burn rehabilitation sessions on pain and range of motion in hospitalized pediatric burn injury patients [24]. The study included 22 participants (13 boys and nine girls), aged 9 to 16 years, with second-degree deep partial thickness burns covering 10%-25% of their TBSA. Through random assignment, researchers divided the subjects into study and control groups. While the control group received a passive range of motion and stretch exercises, the study group underwent the same treatment in conjunction with VR training facilitated by the Oculus Rift DK2. The VR experience allowed participants to choose their preferred videos, creating an immersive gaming environment. Pain intensity was measured using the Visual Analog Scale (VAS), and joint range of motion was quantified using electronic digital goniometer measurements conducted before and immediately following the rehabilitation session. The study's outcomes led to the conclusion that incorporating VR into the rehabilitation program for pediatric burn patients directly affected pain reduction and increased range of motion [24].

## Conclusions

Rehabilitation can help severely burn children since it favours the cardiovascular and respiratory systems, the musculoskeletal system, and body composition. Exercise prescriptions should be thoroughly customized to provide the best possible success in rehabilitation, considering the degree of burns and physical limitations. A patient needs specialized, multidisciplinary care for the best functional and cosmetic outcome after significant burn damage. Clinical guidelines can improve clinical decision-making by offering data and suggestions based on research-based evidence. This case report is intended to serve as a practical manual for the necessary clinical knowledge and therapy intervention approaches for managing burn patients successfully.
